# Origins and Evolutionary Patterns of the *1.688* Satellite DNA Family in *Drosophila* Phylogeny

**DOI:** 10.1534/g3.120.401727

**Published:** 2020-09-15

**Authors:** Leonardo G. de Lima, Stacey L. Hanlon, Jennifer L. Gerton

**Affiliations:** Stowers Institute for Medical Research, Kansas City, Missouri 64110

**Keywords:** Satellite DNA, *1.688*, 359 bp satellite, *Drosophila melanogaster group*, heterochromatin, concerted evolution

## Abstract

Satellite DNAs (satDNAs) are a ubiquitous feature of eukaryotic genomes and are usually the major components of constitutive heterochromatin. The *1.688* satDNA, also known as the 359 bp satellite, is one of the most abundant repetitive sequences in *Drosophila melanogaster* and has been linked to several different biological functions. We investigated the presence and evolution of the *1.688* satDNA in 16 *Drosophila* genomes. We find that the *1.688* satDNA family is much more ancient than previously appreciated, being shared among part of the *melanogaster* group that diverged from a common ancestor ∼27 Mya. We found that the *1.688* satDNA family has two major subfamilies spread throughout *Drosophila* phylogeny (∼360 bp and ∼190 bp). Phylogenetic analysis of ∼10,000 repeats extracted from 14 of the species revealed that the *1.688* satDNA family is present within heterochromatin and euchromatin. A high number of euchromatic repeats are gene proximal, suggesting the potential for local gene regulation. Notably, heterochromatic copies display concerted evolution and a species-specific pattern, whereas euchromatic repeats display a more typical evolutionary pattern, suggesting that chromatin domains may influence the evolution of these sequences. Overall, our data indicate the *1.688* satDNA as the most perduring satDNA family described in *Drosophila* phylogeny to date. Our study provides a strong foundation for future work on the functional roles of *1.688* satDNA across many *Drosophila* species.

Satellite DNAs (satDNAs) consist of tandem repeat sequences typically organized in large arrays in the heterochromatic regions of eukaryotic chromosomes (reviewed by Plohl *et al*. 2012). Regarded as one of the fastest evolving components of the eukaryotic genome (Strachan *et al.* 1985), closely related species may differ dramatically in the number, abundance, and genomic distribution of a given satDNA (Plohl *et al.* 2012; Altemose *et al.* 2014). There are several examples showing that satDNAs are often subject to concerted evolution because the repeat sequences display both a high degree of similarity within a species and a high degree of divergence between species (*e.g.*, Bachmann and Sperlich 1993; Palomeque and Lorite 2008; Pérez-Gutiérrez *et al.* 2012; de Lima *et al.* 2017).

SatDNAs were initially identified in *Drosophila* by their buoyant densities (in g/ml) on cesium chloride gradients to characterize the most abundant sequences in *D. virilis*, *D. melanogaster* and *D. hydei* genomes (Gall *et al.* 1974; Renkawitz 1979). In the past decades, satDNAs have been mostly studied from a small sample of cloned repeats isolated from a limited number of species and individuals. (Brutlag *et al.* 1977; Waring and Pollack 1987; Bachmann and Sperlich 1993) More recently, the usage of genomic approaches has provided novel interesting insights on the origin, organization and evolution of satDNA in *Drosophila* (Larracuente 2014; Dias *et al.* 2014; Khost *et al.* 2017; McGurk and Barbash 2018; Sproul and Khost *et al.* 2020).

In several *Drosophila* species, satDNAs account for more than 30% of the genome. Loss and amplification events of distinct satDNA families are important factors in shaping the architecture and size of the *Drosophila* genome (Bosco *et al.* 2007). Several studies in *Drosophila* point to a biological role for satDNAs, usually related to centromere structure (Sun *et al.* 1997; Henikoff *et al.* 2001) and chromatin organization and modulation (Usakin *et al.* 2007; Gallach 2014). For example, transcripts of the *1.688* satDNA family in *D. melanogaster* contribute to the safeguard mechanism that is required for proper kinetochore complex assembly and accurate chromosomal segregation (Rosic *et al.* 2014). Other findings indicate that siRNAs generated from *1.688* satDNA X-linked euchromatic arrays contribute to the recruitment of the *MSL* complex and seem to play an important role in the dosage compensation mechanisms in *D. melanogaster* (Menon *et al.* 2014). Despite their importance for genome organization, function and evolution, studies focusing on satDNA evolution have been absent in *Drosophila* species with sequenced genomes and in eukaryotes in general.

In *D. melanogaster*, one of the most well-understood satDNAs is the *1.688* g/ml satellite (herein referred to as *1.688* satDNA). This satDNA is also known as 359 bp because the bulk of this satellite is composed of 359 bp repeats present in the heterochromatic portion of the X chromosome (Brutlag 1980); less abundant repeat variants, also known as subfamilies, of 260 bp and 353-356 bp are present on chromosomes 2 and 3, respectively (Losada and Villasante 1996; Abad *et al.* 2000). The *1.688* satDNA is part of the genome of *Drosophila* species from the *melanogaster* subgroup, but its relative abundance may vary more than 40-fold between species (Barnes 1978; Strachan *et al.* 1985; Lohe and Roberts 1988). In addition to the long arrays in heterochromatin, short arrays are present in euchromatic regions of the X chromosome of *D. melanogaster*, *D. simulans*, *D. sechellia* and *D. mauritiana* (Dibartolomeis *et al.*1992; Sproul and Khost *et al.* 2020).

Even though the *1.688* satDNA is one of the best-studied in *Drosophila melanogaster*, only limited information exists for this satDNA in other species from the *melanogaster* subgroup, and none from species outside the subgroup. To rectify this, we took advantage of the wealth of genomic data in several *Drosophila* species to study the *1.688* satDNA in more detail and identify new facets of its evolution, including its origins and evolutionary patterns in heterochromatin and euchromatin. Taken together, our in-depth genomic analysis of 14 *Drosophila* species from both inside and outside the *melanogaster* subgroup is the most comprehensive evaluation of the *1.688* satDNA to date. Our work (1) provides strong evidence that the *1.688* satDNA family is the most broadly maintained satDNA in *Drosophila* phylogeny to date (2) exposes how chromatin domains can impact the sequence evolution of satDNA repeats and (3) suggests potential new functions of this satDNA family.

## Materials and Methods

### Data mining and sequence analysis

*1.688* satDNA repeats were mined from several sequenced species of *Drosophila* by performing BLASTN searches in FlyBase (http://flybase.org/blast) with a collection of *D. melanogaster* subgroup *1.688* satDNA consensus sequences described by Strachan *et al.* (1985). BLASTN searches were restricted to assembled genomes due to the high single-pass error-rates (11–15% for PacBio, similar for Nanopore) (Tørresen *et al.* 2019). The majority of these errors consist of insertion and deletions (indels), leading to misalignments and possible misleading evolutionary analyses.

The Tandem Repeat Finder (Benson 1999) program was used to bolster the data-mining efforts by confirming the monomer size present in each array. The *1.688*-like satDNA arrays found in files representing chromosomes, scaffolds or contigs were manually curated by a dot-plot using *Dotlet applet* (Junier & Pagni 2000; https://dotlet.vital-it.ch/), one by one, to determine the start and end of each repeat, using the *1.688* satDNA consensus sequences of each species or the closest related species as parameters. Sequences were designated as “full repeats” if they had the same start and end relative to our reference *1.688* satDNA repeats. We cannot exclude the possibility that other sequences with homology to *1.688* satDNA exist in the genome of *Drosophila* species but were not retrieved through this methodology.

Aiming to identify new variants of *1.688* satDNA repeats in different species, we used the short sequence cluster method *RepeatExplorer* (http://www.RepeatExplorer.org/) (Novák *et al.* 2013). *RepeatExplorer* identifies repetitive elements *de novo* using a graph-based method to group reads into discrete clusters based on all-by-all blast similarity. This analysis is unbiased and uses a large repertoire of sequences, resulting in a higher quality analysis and more variability of sequences due to its overall sequence clusterization. All reads used in this analysis were generated by *Illumina* whole-genome shotgun reads (Supp. Table 1). Reads were trimmed to 100 bp and sequencing adapters were removed. We excluded reads that were more than 10% below our quality cut-off value of 30. The quality filtering approaches used herein have been described as sufficient to minimize downstream analysis artifacts (Minoche *et al.* 2011). The clusterization threshold was explicitly set to 90% sequence similarity spanning at least 65% of the read length. Only clusters with genomic proportion equal or higher to 0.01% were analyzed in this study, as recommended by the developers of *RepeatExplorer* (Novák *et al.* 2013).

**Table 1:** List of species-specific sets of primers used to amplify *1.688* satDNA tandem repeated arrays in nine *Drosophila* species

SPECIES	FWD	REV
***D. MELANOGASTER***	5`-cgttagcactggtaattagctgc-3`	5`-cgatccctattactttttgaagg-3`
***D. SIMULANS/D. SECHELLIA***	5`-gtttgtttcttaaatcccaatcg-3	5`-ctcaacgaggtatgacattcc-3`
***D. ERECTA***	5`-gccggatgttttaggaggtt-3`	5`-aggtatggcattccactcttggac-3`
***D. YAKUBA***	5′-ccatacctcgttgaattcg-3`	5`-cattccactttggcaac-3`
***D. EUGRACILIS***	5`-tcatacatcgatgaactcgt-3`	5`-cattccatagtccgacaa-3`
***D. BIARMIPES***	5`-ccaataaattggcatcaa-3`	5`-cgagctcagcaaggtatgaca-3`
***D. TAKAHASHII***	5`-cttaattctcaatcgatttgc-3`	5`- ctacgagctcaacaaggta-3`
***D. SUZUKII***	5`-ggctaaacaacgactga-3`	5`-aatcaaggcgtacagctaa-3`

### Assignment of repeats to heterochromatin and euchromatin

Our *1.688*-like satDNA repeats were assigned to heterochromatin or euchromatin based on analyses of the flanking sequences within 0.5 to 2 kb, as previously described (Kuhn *et al.* 2012). We searched for flanking sequences using BLASTN against each species genome and manually checked them in GBrowse (http://flybase.org/cgi-bin/gbrowse2). The presence of transcriptionally active genes in genomic previously described as euchromatic in *D. melanogaster* and their orthologs in *D. simulans*, *D. sechellia*, *D. erecta* and *D. yakuba* was taken as supporting evidence of euchromatic location of repeats, and similarly for the *melanogaster* subgroup using features in *FlyBase* (http://flybase.org). Phylogenetic clusters were used to additionally separate the groups of sequences based on similarity to those previously described within euchromatic regions. *1.688* satDNA repeats derived from heterochromatin and euchromatin differ by ∼30% and tend to form distinct phylogenetic clusters (Kuhn *et al.* 2012; de Lima *et al.* 2017), therefore for species that lack annotated genomes we relied solely on phylogenetic clusterization to assign arrays. Multiple sequence alignments were performed using Muscle 4.0 (Edgar 2004) with default options. Incomplete, partial or truncated monomers (< 75% of the expected monomer size) were not used in the interspecific analyses. We identified the best substitution model with jModelTest2 (Darriba *et al.* 2012) for each alignment, which was subsequently used for phylogenies and distance matrices. The optimal model tended to be Kimura 2-parameters/T93 models with gamma distribution. The MEGA software version 7.1 (Tamura *et al.* 2014, Kumar *et al*. 2016) was used for the calculation of genetic distances and construction of Neighbor-Joining (NJ) dendrograms and *Tajima’s D* test calculations.

The identification of conserved motifs in *1.688* satDNA derived from heterochromatin was carried out using the software MEME (http://meme.sdsc.edu) (Bailey *et al.* 2009). We searched for motifs of 10 - 200 bp that had statistical values lower than 10^−6^.

### Genomic distribution on assembled genomes of D. melanogaster, D. simulans and D. yakuba

To map the distribution of *1.688* satDNA arrays in euchromatin, we retrieved assembled files of chromosomes 2L, 2R, 3L, 3R and X from *D. melanogaster*, *D. simulans* and *D. yakuba* present on *FlyBase* (www.flybase.org). We derived a chromosome-specific consensus sequence and used each one as a query for local BLASTN searches (Altschul *et al*. 1990) with the corresponding chromosome and species files. To calculate density, the position of the hit and the amount of *1.688* satDNA (in bps) was plotted in non-overlapping 100 kb intervals. Data were plotted using software R version 3.2.2 (R Core Team 2015).

To identify the presence of *1.688* satDNA sequences in the vicinity of genes we used the *Table Browser* genomic tool implemented in the UCSC *GenomeBrowser* (www.genome.ucsc.edu) for the annotated genomes of *D. melanogaster*, *D. simulans*, *D. sechellia*, *D. erecta* and *D. yakuba*. We isolated genic and intronic sequences, along with 5 kb up and downstream for all annotated genes in all five species. Each of these data sets was independently queried with local BLASTN searches using the species-specific *1.688* satDNA consensus sequence. We used as a cutoff level 65% of identity, 70% of coverage and e-value <10^−05^ in order to prevent non-specific results. With the exception of *D. yakuba*, we did not have redundancies in our queries despite *Drosophila* genomes having a high gene density (Hou *et al.* 2012): of the 792 initial hits in *D. yakuba*, only ten hits were found in both the upstream and downstream query results (all intronic hits were unique).

#### Fly stocks:

All fly stocks were raised on standard Bloomington medium at 25°, and male and female third instar wandering larvae were used. The following fly stocks were used: *D. melanogaster* (Oregon R), *D. simulans w501* (DSSC#14021-0251.195), *D. sechellia* (DSSC#14021-0248.01), *D. erecta* (DSSC#14021-0224.01), *D. yakuba* (DSSC#14021-0261.01), *D. eugracilis* (DSSC#14026-0451.02), *D. biarmipes* (DSSC#14023-0361.06), *D. takahashii* (DSSC#14022-0311.10). *D. suzukii* specimens were identified and collected by Prof. Jennifer Gleason in Missouri and Kansas locations during the years of 2017 and 2018. *D. suzukii* flies were cultivated by Prof. Jennifer Gleason at the Ecology and Evolutionary Biology Department at University of Kansas, Lawrence, in accordance with FDA regulations.

Genomic DNA was extracted from 30 frozen flies using the Promega Maxwell 16 robot protocol and its respective extraction kit (www.promega.com/-/media/files/resources/protocols/technical-manuals/0/maxwell-16-instrument-as1000-operating-manual.pdf). Species-specific primer sets were designed to amplify highly conserved regions identified from alignment data (Table 1). PCR reactions consisted of an initial denaturation step of 94° for 3 min, followed by 30 cycles of 94° for 60 sec, 55° for 60 sec, and 68° for 60 sec, and then a final extension at 68° for 10 min. PCR products were excised from 1% agarose gels and purified with the IBI Gel Extraction Kit. Probes were labeled with 16-dUTP-biotin by nick-translation according to the Enzo Life Sciences’ Nick Translation DNA Labeling System.

### Preparation, DNA staining and FISH on mitotic and polytene chromosome spreads

Mitotic chromosome spreads from larval brain tissue were prepared as previously described in detail (Hanlon *et al.* 2018). The brain from a single third-instar larvae was dissected in 0.7% sodium chloride and moved to a fresh 50 µl drop of 0.5% sodium citrate for hypotonic treatment for 5 min, followed by fixation for 4 min in 2 ml of fixative solution (45% acetic acid, 2.5% paraformaldehyde). After fixation, the brain was transferred to a 3 µl drop of 45% acetic acid on an 18-mm × 18-mm siliconized coverslip, gently squashed with an inverted microscope slide, then heavily squashed for 2 min using a hand clamp. The slide was then immediately placed into liquid nitrogen for at least 5 min, after which the coverslip was popped off the slide using a razor blade, dehydrated in 70% ethanol for at least 10 min at −20°, then transferred to 100% ethanol at −20° for at least 10 min. Each slide was air-dried and 21 µl of the FISH solution (20 ul 50% formamide, 10% dextran sulfate, 2× SSC plus 1 ul of biotinylated probe, see above) was applied directly to the sample area and a clean 22 mm × 22 mm glass coverslip was placed on top. The slide was heated to 95° on a heat block for 5 min in darkness, then transferred to a hybridization chamber consisting of a container lined with damp paper towels (to maintain humidity and prevent the samples from drying out) at 30° for overnight (16–24 h).

After the incubation, slides were washed three times in 0.1x SSC for 15 min each, then put in blocking solution (4x SSC, 3% BSA, 0.1% Tween-20) at 37° for 30 min. Excess blocking solution was wiped from around the sample area, followed by application of the secondary solution (40 μl 4x SSC, 1% BSA, 0.1% Tween-20 plus 0.8 μl avidin 488 (Roche) antibody). Each slide was covered with a 22 mm × 22 mm coverslip then put into a hybridization chamber at 37° for 30 min. Slides were washed three times in SSCT (4x SSC, 0.1% Tween-20), followed by three washes in 0.1x SSC; each wash was five minutes. Excess liquid was wiped from around the sample area, and each slide was mounted with 5 μl Vectashield+DAPI and a 22 mm × 22 mm coverslip. Coverslip edges were sealed with nail polish and imaged immediately.

Polytene chromosomes FISH experiments were conducted as described in de Lima *et al.* (2017). Briefly, salivary glands from third stage larvae were dissected on 0.7% NaCl solution and transferred immediately to 1X PBS solution. Next, the salivary glands were fixed in Carnoy solution (3:1 ethanol/acetic acid) and then moved to a 50-µl drop of 1:1 lactic acid/acetic acid for 5 min and squashed. The slide was incubated at room temperature for two hours and then immediately placed into liquid nitrogen for at least 3 min. Immediately after removal, the coverslip was popped off the slide using a razor blade, and dehydrated at room temperature in 100% ethanol for at least 10 min. For FISH, each dried slide was fixed with paraformaldehyde 4% solution for 30 min and then transferred to 100% ethanol. Slides were then incubated in 2xSSC at 65° for 30 min and dehydrated in 70% and 96% ethanol for 10 min each. Chromosomes were denatured in 0.07 M NaOH solution for 30 sec and immediately incubated in 2XSSC solution for 10 min. Slides were then dehydrated in two consecutive 2 min incubations of 70% and 96% ethanol. For each slide, 20 µl of the FISH solution (50% formamide, 10% dextran sulfate, 2× SSC, 100 ng fluorescence-labeled probe) was applied directly to the dried slide and a clean Parafilm 30 mm × 30 mm coverslip was placed on top. Slides were incubated at 37° overnight (16–24 hr), and washed twice in 2XSSC at 37° for 5 min. The probes were detected with avidin 488 (Roche) then mounted by applying 5 µl Vectashield with DAPI to a clean 22-mm × 22-mm no. 1.5 glass coverslip.

### Microscopy and image processing

Mitotic chromosome images were acquired with a DeltaVision microscopy system (GE Healthcare, Piscataway, NY) consisting of a 1 × 70 inverted microscope with a high-resolution charge-coupled device (CCD) camera. Polytene chromosome images were acquired with an Inverted Zeiss LSM 780 confocal microscope. All imaging used a 63x objective for polytene and 100x objective with 1.6 auxiliary magnification for mitotic chromosomes. Stacks of deconvolved images were combined in a *z*-projection showing maximum intensity, cropped to the region of interest, recolored, and adjusted for brightness and contrast in FIJI/ImageJ.

### Data availability

Original data underlying this manuscript can be accessed from the Stowers Original Data Repository at http://www.stowers.org/research/publications/libpb-1549. All stocks (except *D. suzukii*) and reagents available upon request. Supplemental material available at figshare: https://doi.org/10.25387/g3.12952253.

## Results

**Figure 1:**
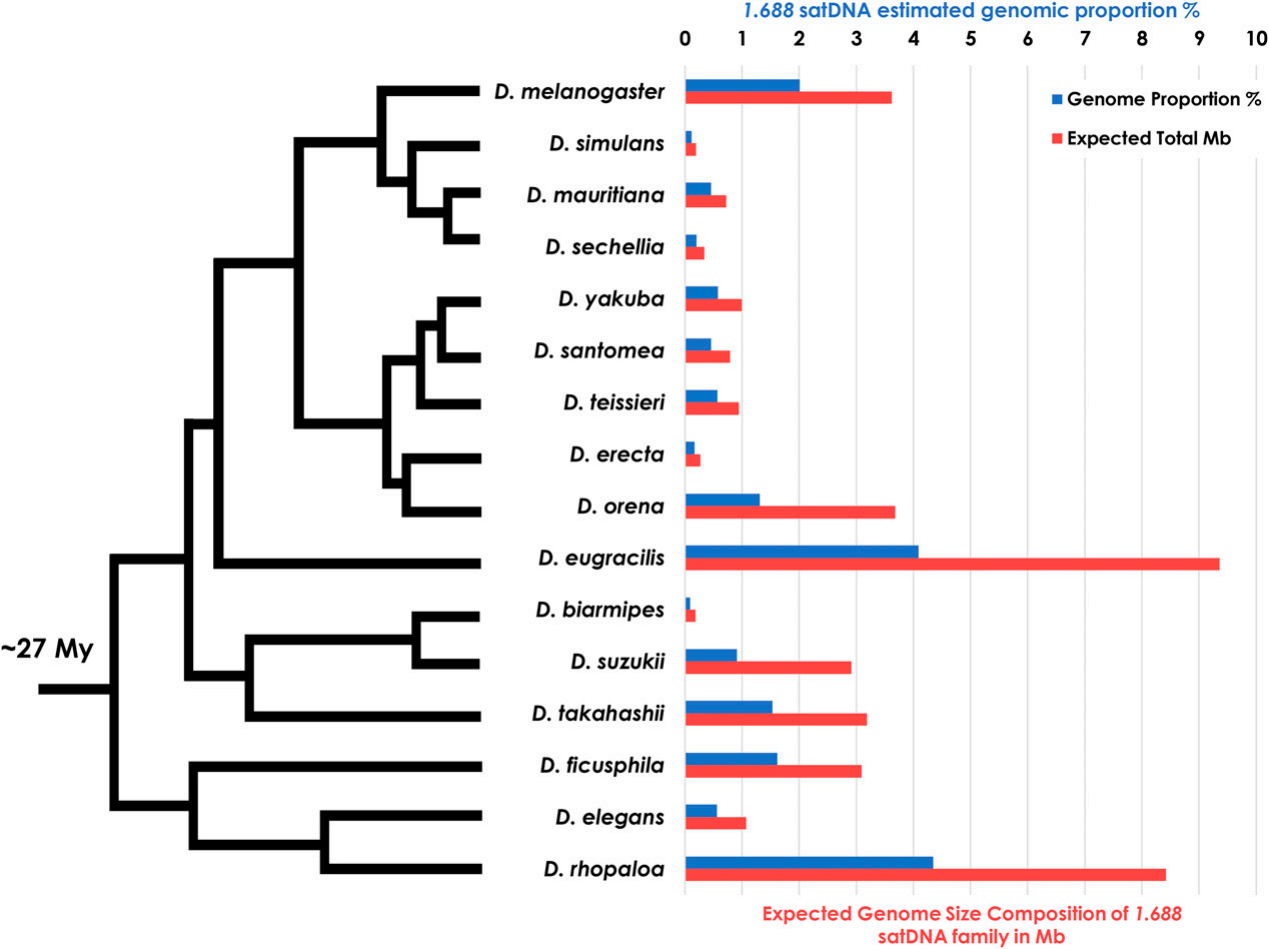
Variation in the genomic proportion of the *1.688* satellite DNA family throughout *Drosophila* phylogeny. Bars indicate the estimated genomic proportion (blue) and the total amount of DNA in Mb (red) comprised by the *1.688* satDNA family based on the *RepeatExplorer* analysis for each of the 16 species of *Drosophila* analyzed. Phylogeny adapted from Russo *et al.* (2013).

To broadly identify satDNA sequences across the *Drosophila* phylogeny belonging to the *1.688* satDNA family, we performed BLAST searches using a collection of *D. melanogaster 1.688* consensus sequences (Strachan *et al.* 1985) against all available *Drosophila* sequenced genomes to date. We found 16 species that carried *1.688* satDNA repeats, 14 of which have fully assembled genomes (Figure 1). As expected, we found *1.688* satDNA repeats present in all five sequenced species from the *melanogaster* subgroup, *i.e.*, *D. melanogaster*, *D. simulans*, *D. sechellia*, *D. erecta* and *D. yakuba*, but absent in the genomes of other species from the *melanogaster* group including *D. kikkawai*, *D. ananassae* and *D. bipectinata*, and several other more phylogenetically distant species such as *D. pseudoobscura*, *D. miranda* and *D. persimilis* (*obscura* group), *D. mojavensis* (*repleta* group) and *D. virilis* (*virilis* group). The usage of the *RepeatExplorer* short sequence *de novo* cluster method in this study aimed to minimize the underrepresentation of previous estimations of satDNAs and other repetitive families calculated in *Drosophila* by means of genome assemblies (Drosophila 12 genomes Consortium *et al.* 2007). After applying a stringent quality cutoff, the reads used in the overall *de novo* clusterization had genome coverage of at least 0.55-fold (Table S1), suggesting that most, if not all, repetitive sequences were clustered and analyzed in this study (See Methods). We retrieved a total of 10,855 monomers of *1.688* satDNA repeats from the assembled genomes of 14 species with evidence for significant abundance in the genomes of *D. melanogaster* subgroup species as well as *D. eugracilis*, *D. suzukii*, *D. biarmipes*, *D. takahashii*, *D. ficusphila*, *D. elegans*, and *D. rhopaloa* (Table 2). These species shared a common ancestor ∼27 Mya, making the *1.688* satDNA the most broadly maintained satDNA sequence in *Drosophila* phylogeny described to date. The genomic contribution varied significantly among the species, especially in D. elegans and D. rhopaloa, which show a greater genomic proportion when compared to the D. melanogaster subgroup (Figure 1). Notably, we did not observe any satDNA family with similar characteristics in *D. kikkawai* or any other more phylogenetically distant species (*D. leontia*, *D. ananassae* and *D. bipectinata*), either in assembled genomes or *RepeatExplorer* outputs -that were analyzed with a similar genomic coverage (Table S1). Therefore, the presence of *1.688* satDNA sequences in the *rhopaloa* subgroup and absence in *D. kikkawai*, *D. leontia* (*montium subgro**up*) and *D. ananassae* and *D. bipectinata* (*ananassae subgro**up*) suggests that the *1.688* satDNA family either originated or expanded before the *D. melanogaster-D. rhopaloa* clade diversification that occurred approximately 27 Mya.

**Table 2:** Number of *1.688* satDNA copies analyzed in each *Drosophila* genome and characterized as heterochromatic or euchromatic (see Methods)

	Heterochromatic copies	Euchromatic copies	*1.688* satDNA subfamilies (bp)
***D. melanogaster***	1860	168	360 /353 /257
***D. simulans***	1568	129	360 /199
***D. sechellia***	951	144	361 /198
***D. mauritiana***	829	102	361 /198
***D. erecta***	297	595	361 /198
***D. orena***	1473	42*^a^*	360 /198
***D. santomea****^b^*	—	—	191
***D. teissieri****^b^*	—	—	191
***D. yakuba***	77	930	360 /191
***D. eugracilis***	95	63	358
***D. biarmipes***	121	73	360
***D. suzukii***	244	35	350 /198
***D. takahashii***	59	47	337
***D. ficusphila***	61	18	197
***D. elegans***	52	45	390
***D. rhopaloa***	347	51	365 /189

a *D. orena* euchromatic copies identified were mostly truncated or partial when compared to heterochromatic copies.

b *D. santomea* and *D. teissieri* do not have assembled genomes. *1.688* satDNA sequences analyzed were retrieved from assembled contigs generated as output of *RepeatExplorer* pipeline.

### A new 1.688 subfamily of ∼190 bp is present in 11 species of Drosophila

**Figure 2:**
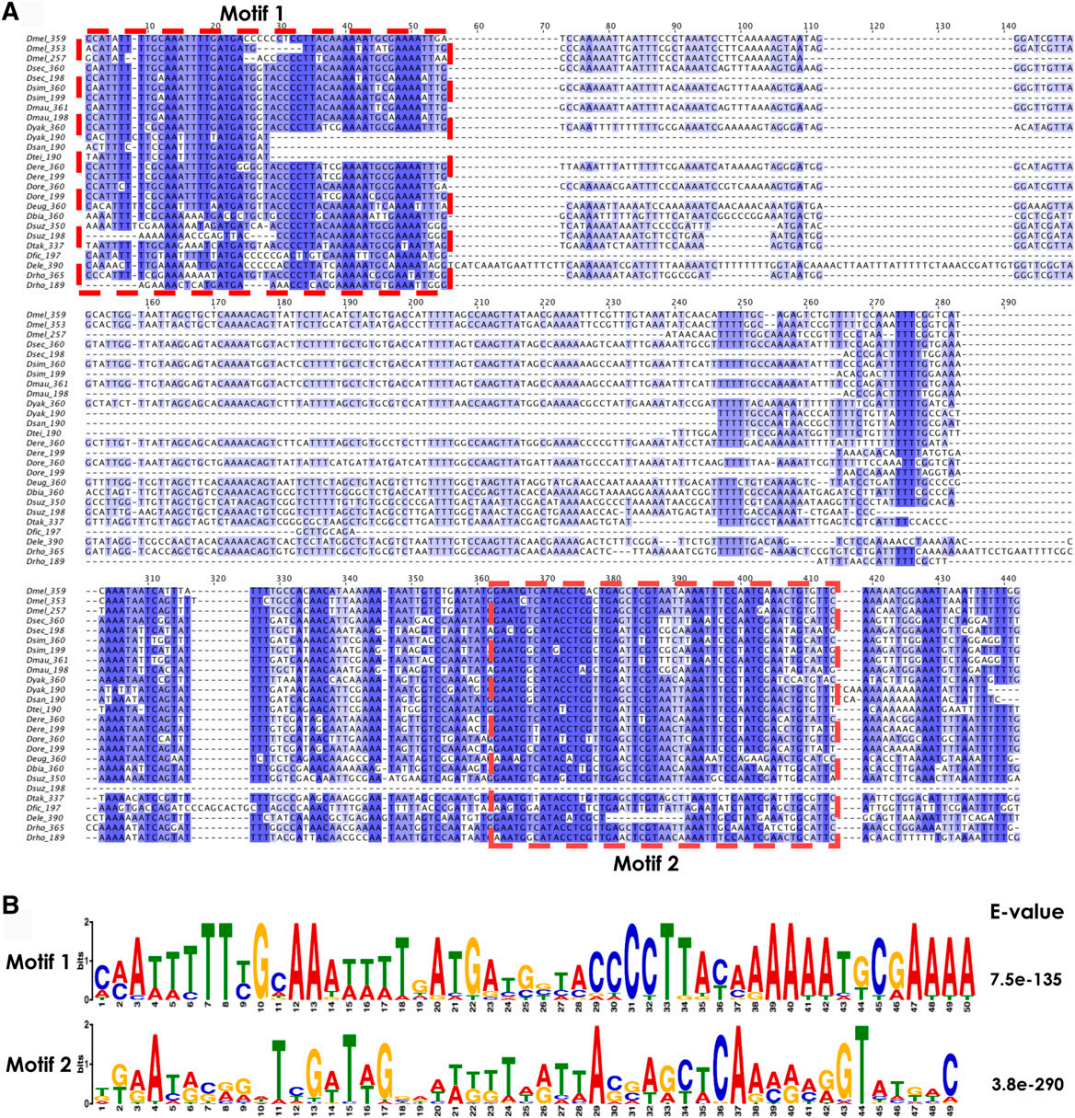
*1.688* satDNA sequence analysis for repeats derived from heterochromatin. A. Consensus sequences for subfamilies from each of the 16 species were aligned by species. The subfamily description is denoted on the left for each monomer. Dark blue indicates regions with high sequence conservation whereas white regions indicate no conservation among the sequences. B. Two conserved motifs within the 360 bp subfamily were identified by MEME and are shared by all 16 species of *Drosophila*.

The most common monomer size for the *1.688* satDNA repeat in most species is ∼360 bp, but in our sequence analysis we identified a new variant (subfamily) of ∼190 bp present throughout *Drosophila* phylogeny. Previous studies have described two chromosome-specific subfamilies of ∼260 bp and 353 bp on chromosomes 2 and 3, respectively, in *D. melanogaster* (Losada and Villasante 1996; Abad *et al.* 2000), and a 196 bp monomer has been previously described by Strachan *et al.* (1985) in *D. teissieri*, indicating that *1.688* satDNA monomer size is not restricted to ∼360 bp. In fact, the 190 bp subfamily is a common *1.688* satDNA variant present in 11 of the 16 species we analyzed, similar to the range of species in which the 360 bp subfamily is found (Table 2). Also, like the 360 bp variant, the number of ∼190 bp monomers was variable in each species (Table S2). When the monomers from the 190 bp subfamily were aligned alongside the 360 bp subfamily, two striking features emerge. First, monomers from the 190 bp subfamily share a common deletion of ∼170 bp in the central portion when compared to the 360 bp consensus (Figure 2A and Figure S1). Second, both subfamilies share two well-conserved motifs, referred to as Motif 1 and Motif 2 (Figure 2B). The presence of these two conserved regions shared among *1.688* satDNA sequences from different species may indicate functional constraints, possibly related to protein-binding sites (Ugarkovic 2005). Evolutionary conservation can be selected based on primary sequence or the ability to form non-canonical secondary or tertiary structures needed for functional interactions. Further experimentation will be required to fully understand the significance of the motifs. *D. suzukii* is notable because the 190 bp subfamily is still ∼190 bps, but does not share the same deletion compared to the other species (Figure 2 and Figure S1). The *D. suzukii* 198 bp monomer has Motif 1 and most of the central region that is conserved in the 360 bp subfamily monomers but does not carry Motif 2 and its surrounding sequence (Figure 2). Based on these findings we hypothesize that the 190 bp subfamily is a derivative of the 360 bp subfamily, produced from an event that occurred independently prior to the *D.melanogaster-D.rhopaloa* clade diversification (see Discussion).

### Concerted evolution of 1.688 satDNA in heterochromatin and enrichment on the X chromosome

We identified 10,855 copies of *1.688* satDNA repeats (Table 2). Since *1.688* satDNA has been shown to be present in both heterochromatin and euchromatin, we used two approaches to computationally assign each individual copy as being heterochromatic or euchromatic following established protocols (Kuhn *et al.* 2012). Analyses of flanking sequences within 0.5 to 2 kb, when available, together with the methodology of phylogenetic tree clusterization of heterochromatic and euchromatic copies enabled us to assign 8,034 repeats as heterochromatic, while the other 2,821copies were inferred as euchromatic (see Methods). As a result of this analysis, we determined the proportion of heterochromatic and euchromatic copies among the 14 species we studied with assembled genomes: *D. melanogaster*, *D. simulans*, *D. sechellia*, *D. mauritiana*, *D. orena*, *D. erecta*, *D. yakuba*, *D. eugracilis*, *D. biarmipes*, *D. suzukii. D. takahashii*, *D. ficusphila*, *D. elegans* and *D. rhopaloa*. (Figure S2). With the exception of *D. erecta* and *D. yakuba*, each of the species we analyzed have a higher abundance of heterochromatic copies than euchromatic copies. The overall abundance of *1.688* satDNA sequences identified and characterized may differ among the species analyzed due to differences in genomic assembly efforts. However, the pattern observed is consistent with previous models that predict an increased accumulation of repetitive sequences in chromosomal regions with low recombination, such as heterochromatin (Charlesworth *et al.* 2005).

**Figure 3:**
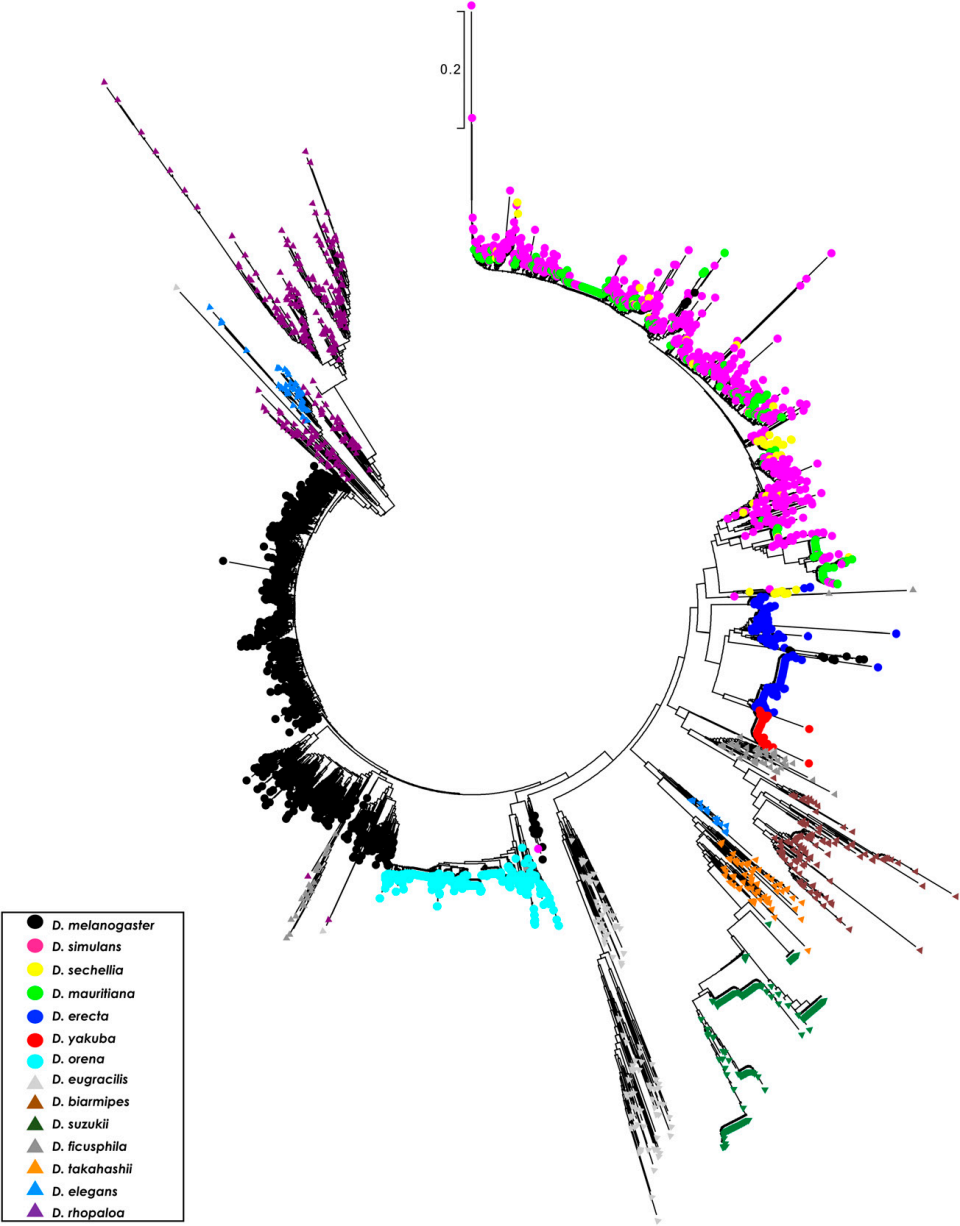
Clusterization reveals species specificity, indicative of concerted evolution in *1.688* satDNA heterochromatic arrays. An unrooted phylogenetic tree using 4111 full-length *1.688-like* monomers derived from heterochromatin from 14 species of *Drosophila* shows a species-specific pattern throughout the *melanogaster* group phylogeny. The evolutionary history was inferred using the Neighbor-Joining method. The tree is drawn to scale, with branch lengths in the same units as those of the evolutionary distances used to infer the phylogenetic tree. The evolutionary distances were computed using the Kimura 2-parameter method and are in the units of the number of base substitutions per site. The rate variation among sites was modeled with a gamma distribution (shape parameter = 1). All positions with less than 85% site coverage were eliminated, *i.e.*, fewer than 15% alignment gaps, missing data, and ambiguous bases were allowed at any position.

To better understand the evolutionary relationships among *1.688* satDNA family monomers, we ran a Neighbor-Joining (NJ) tree constructed with 4,111 full-length heterochromatic *1.688* satDNA repeats extracted from the sequencing reads of the 14 species with assembled genomes. We restricted the analysis to full-length copies to avoid biased alignments originating from partial or truncated sequences (see Methods). The clusterization produced trees with branches that contained repeats from a single species (*e.g.*, *D. melanogaster*) or phylogenetically very closely related species (*e.g.*, *D. simulans*, *D. mauritiana* and *D. sechellia*) (Figure 3). This preferential clusterization in a species-specific pattern indicates a concerted mode of evolution, *i.e.*, repeats within each species are more similar to each other than to repeats between species. Our results also recapitulated the chromosome-specific clustering pattern for *1.688* satDNA in *D. melanogaster* present in chromosomes X, 2 and 3, (Kunh *et al.* 2012; Khost *et al.* 2017) (Figure S3A), indicating intrachromosomal concerted evolution. A similar pattern was observed in *D. erecta*, *D. suzukii*, *D. elegans* and *D. rhopaloa* suggesting chromosomal- or array-specific evolution of *1.688* satDNA in these species (Figures S3B-D). In contrast, *D. simulans*, *D. mauritiana* and *D. sechellia* formed a single large branch that lacked species-specific clusters. This may be the result of the close phylogenetic relationship of these species (Garrigan *et al.* 2012), reinforcing the idea that concerted evolution is a time-dependent mechanism (Strachan *et al.* 1985).

The outcome of the NJ analysis is congruent with the species-specific nucleotide divergence observed in the phylogenetic analysis (Table 3). The interspecific variation among *1.688* satDNA repeats exceeds 21% on average, except between *D. simulans*, *D. mauritiana* and *D. sechellia* that diverge only ∼8% from each other, and *D. orena* and *D. melanogaster* in which the copies show an 18.2% nucleotide divergence. Intraspecific values trended much lower than interspecific values and varied from 2% in *D. yakuba* to 31% in *D. eugracilis* (Table 3). In summary, the evolutionary pattern of the *1.688* satDNA family is incongruent with the phylogenetic relationships for the *melanogaster* group (Figure 3; Figure S4), consistent with the idea that satDNA families are good taxonomic markers (Picariello *et al.* 2002), but do not reflect the phylogenetic history of the *D. melanogaster-D. rhopaloa* clade.

**Table 3:** Intra- and interspecific nucleotide divergence (*p*-distance) of 4,111 *1.688* satDNA heterochromatic copies retrieved from 14 species of *Drosophila*

	*D. mel*	*D. sim*	*D. sec*	*D. mau*	*D. ere*	*D. yak*	*D. ore*	*D. eug*	*D. bia*	*D. suz*	*D. tak*	*D. fic*	*D. ele*	*D. rho*
***D. mel***	0.100													
***D. sim***	0.261	0.080												
***D. sec***	0.251	0.084	0.080											
***D. mau***	0.257	0.079	0.079	0.070										
***D. ere***	0.264	0.306	0.300	0.301	0.130									
***D. yak***	0.264	0.299	0.297	0.293	0.202	0.020								
***D. ore***	0.182	0.260	0.256	0.257	0.280	0.268	0.070							
***D. eug***	0.324	0.317	0.316	0.316	0.380	0.372	0.341	0.310						
***D. bia***	0.304	0.343	0.333	0.334	0.369	0.343	0.323	0.392	0.150					
***D. suz***	0.349	0.419	0.415	0.410	0.366	0.394	0.330	0.434	0.400	0.100				
***D. tak***	0.307	0.341	0.335	0.332	0.340	0.329	0.314	0.399	0.332	0.276	0.190			
***D. fic***	0.223	0.241	0.237	0.239	0.262	0.246	0.256	0.359	0.270	0.356	0.285	0.140		
***D. ele***	0.286	0.306	0.300	0.299	0.349	0.357	0.302	0.392	0.373	0.399	0.338	0.312	0.200	
***D. rho***	0.258	0.351	0.353	0.356	0.353	0.372	0.293	0.390	0.348	0.402	0.392	0.310	0.356	0.21

**Figure 4:**
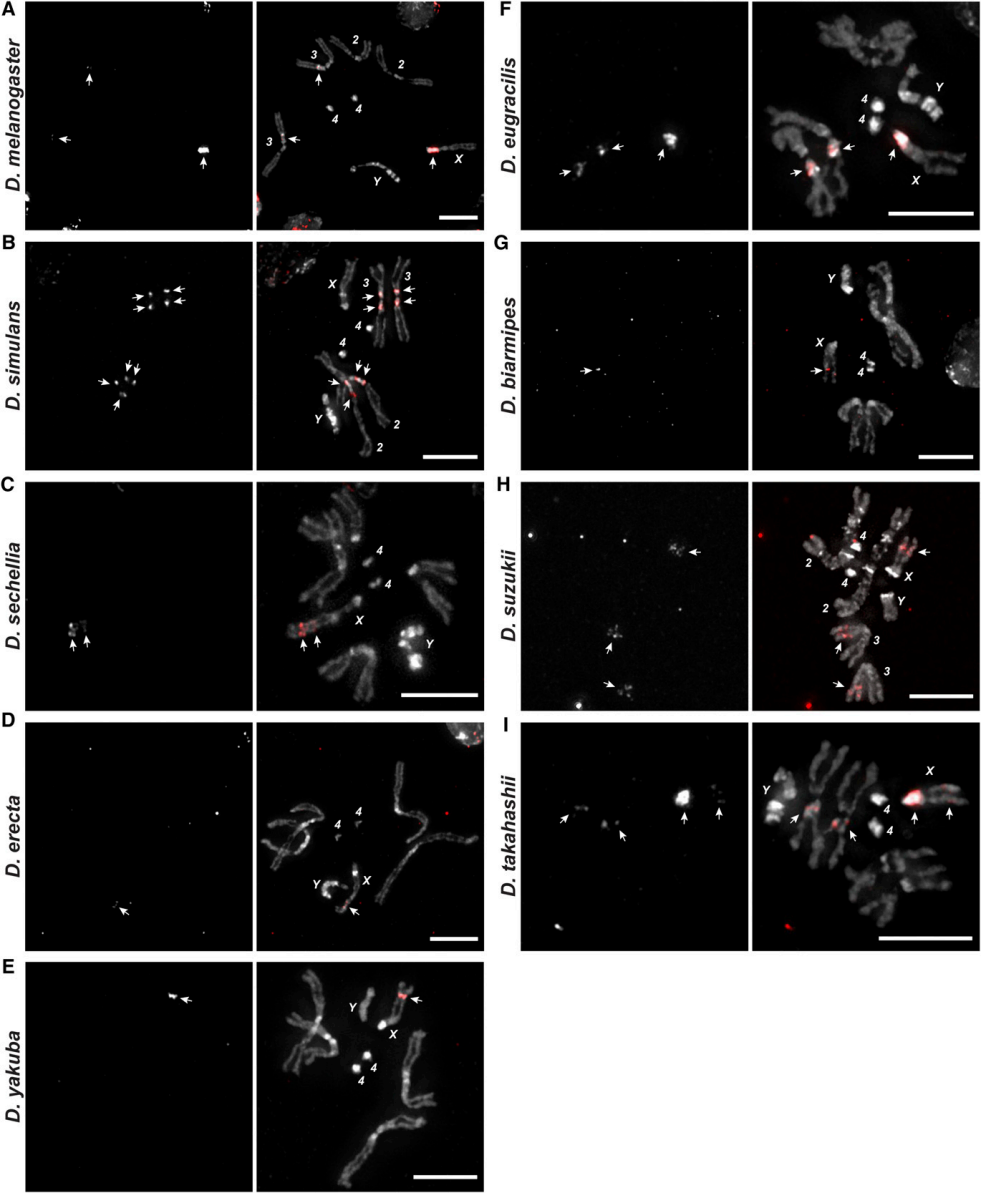
Differential and shared locations of *1.688* arrays in heterochromatin visualized in nine *Drosophila* species from the *melanogaster gro**up*. FISH was performed on neuroblast chromosome spreads from *D. melanogaster*, *D. simulans*, *D. sechellia*, *D. erecta*, *D. yakuba*, *D. eugracilis. D. biarmipes*, *D suzukii* and *D. takahashii* (A-I, respectively). The panels show species-specific probes for 1.688 satDNA (red) labeled with Bio-16-dUTP combined with DAPI staining (gray). The X and Y sex chromosomes and autosomal labels (when known) are identified in each panel. Note that the X chromosome from all species except *D. simulans* has a clear signal. Bar = 5 µm. White arrows indicate signals of hybridization.

**Figure 5:**
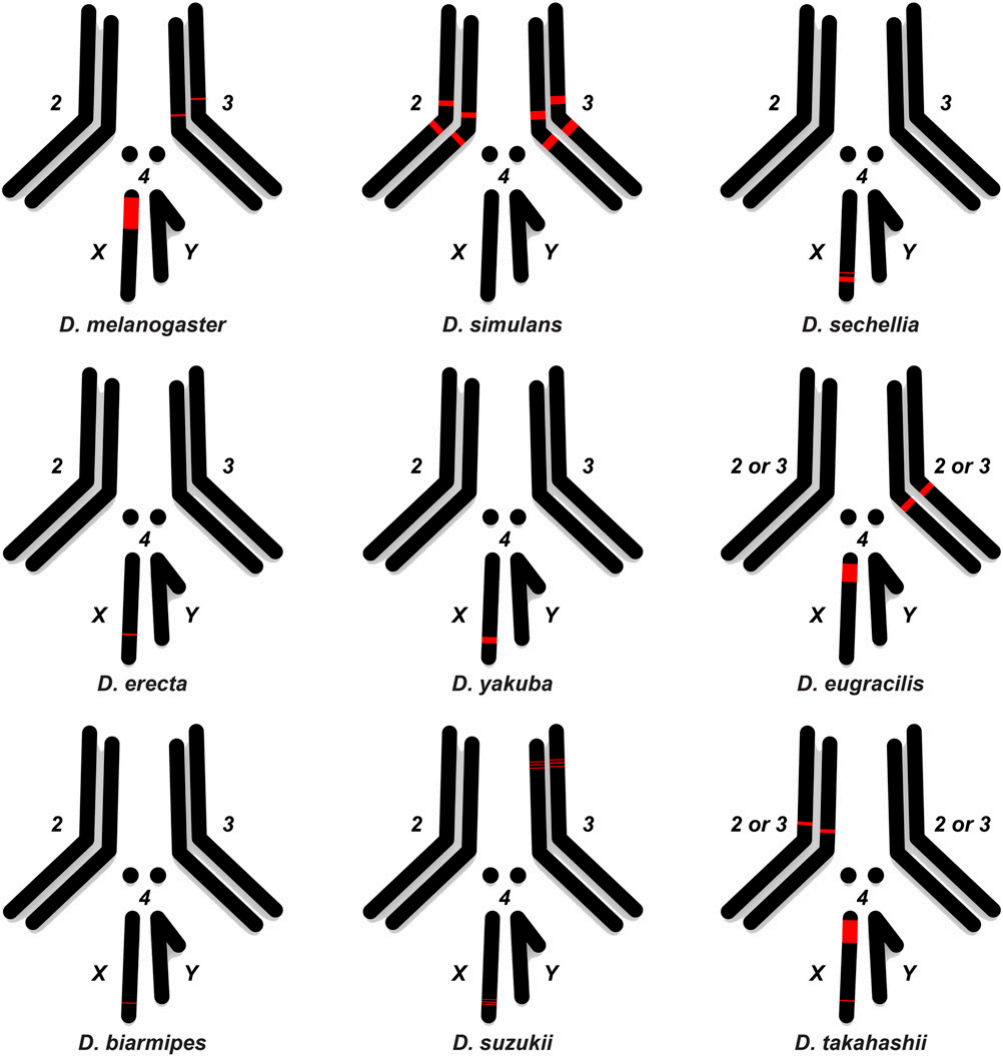
Ideogram showing chromosomal location of *1.688* heterochromatic arrays mapped by FISH in nine *Drosophila* species from the *melanogaster gro**up*. Simplified karyotypes (black) from *D. melanogaster*, *D. simulans*, *D. sechellia*, *D. erecta*, *D. yakuba*, *D. eugracilis. D. biarmipes*, *D suzukii* and *D. takahashii* are shown along with the approximate location and relative abundance of *1.688* satDNA (red) for each species. Note that although chromosomes may have different sizes in each species, the ideograms are similarly sized for the sake of simplicity. The *1.688* satDNA arrays on chromosome 2L in *D. melanogaster* described by Abad *et al.* (2000) were not detected with the probes in this study (see Table 1).

To confirm and extend our computational analysis, we experimentally mapped the distribution of the *1.688* satDNA family throughout *Drosophila* species by performing fluorescent *in-situ* hybridization (FISH) experiments using species-specific probes on the mitotic chromosomes of squashed larval brains from *D. melanogaster*, *D. simulans*, *D. sechellia*, *D. erecta*, *D. yakuba*, *D. eugracilis*, *D. biarmipes*, *D. suzukii* and *D. takahashii* (Figure 4). We observed dramatic variation in abundance that corroborates the predictions from *RepeatExplorer* (Figure 1). All species except *D. simulans* had *1.688* satDNA repeats on the X chromosome. Additionally, we observed a signal on the distal portion of the X chromosome in *D. sechellia*, *D. erecta*, *D. yakuba*, *D. biarmipes*, *D. suzukii*, and *D. takahashii* that had a similar chromosomal location but differed in intensity. This distal X chromosome signal was the only detectable signal observed in *D. sechellia*, *D. erecta*, *D. yakuba*, *D. biarmipes*, suggesting that the region could be the initial point of amplification of this satDNA family. *1.688* satDNA arrays were also present at different sites and on different chromosomes (Figure 5), although the precise determination of autosome (second or third) for *D. eugracilis* and *D. takahashii* was not possible (Figures 4 and 5). Taken together, our data provide striking evidence that, despite being conserved for ∼27 My, the *1.688* satDNA sequences show significant differences in abundance, sequence evolution, and chromosomal distribution in *Drosophila* species.

### 1.688 satDNA is spread throughout euchromatic regions in the D. melanogaster-D. rhopaloa clade

**Figure 6:**
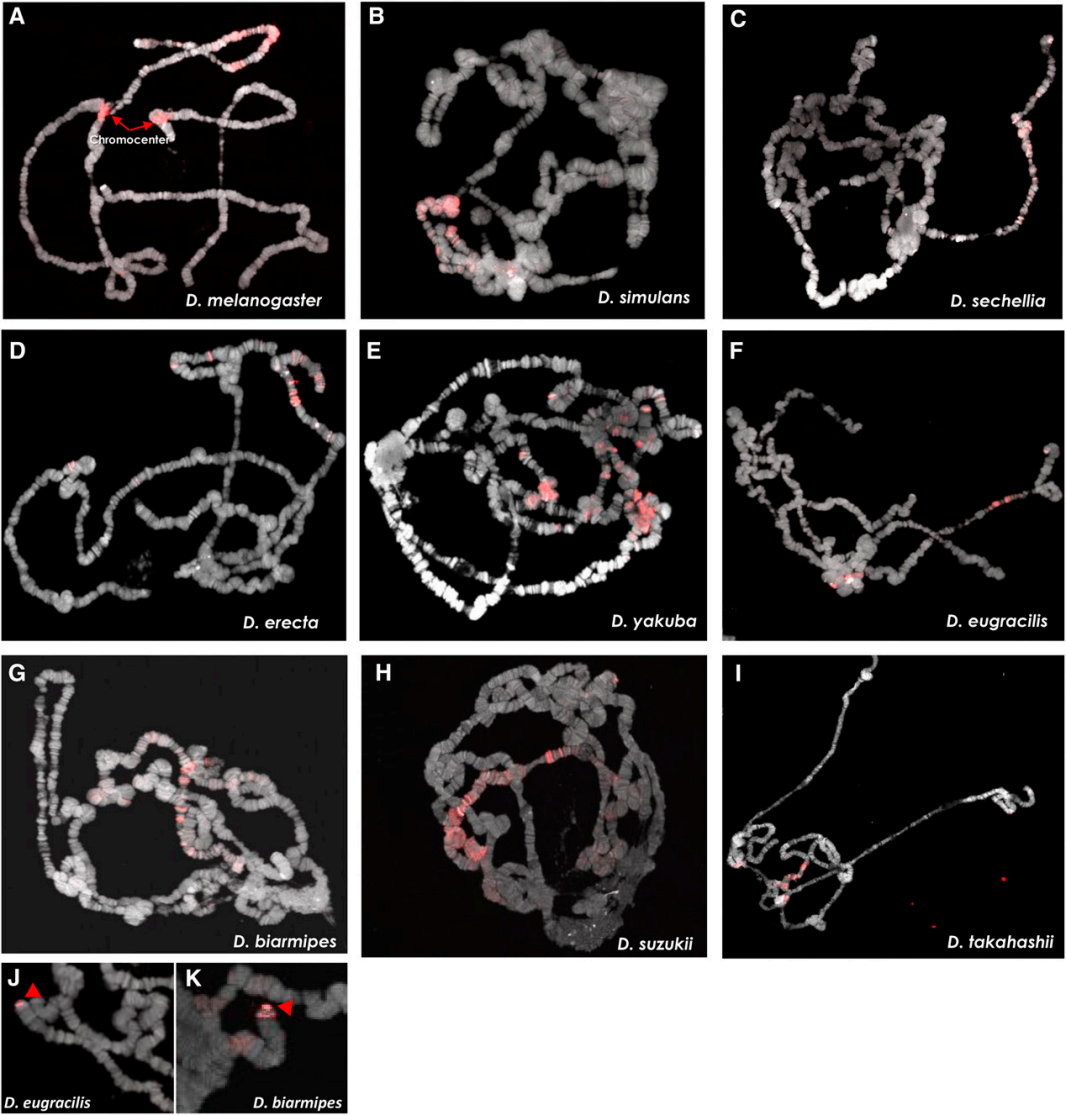
Localization of *1.688* arrays in euchromatin of polytene chromosomes for nine *Drosophila* species from the *melanogaster* group. FISH using species specific probes was performed on salivary gland polytene chromosome from *D. melanogaster*, *D. simulans*, *D. sechellia*, *D. erecta*, *D. yakuba*, *D. eugracilis. D. biarmipes*, *D suzukii* and *D. takahashii* (A-I, respectively). The panels show probes (red) labeled with Bio-16-dUTP combined with DAPI staining (gray). J-K. FISH indicating telomeric/subtelomeric *1.688* satDNA signals of hybridization (red arrowheads) on *D. eugracilis* and *D. biarmipes* chromosomes.

With our methodology, we identified 2,821 putative euchromatic copies of *1.688* satDNA in the 14 species with assembled genomes. This is the first report of *1.688*-like satDNA copies in the euchromatin of *D. erecta*, *D. yakuba*, *D. eugracilis*, *D. biarmipes*, *D. suzukii*, *D. takahashii*, *D. ficusphila*, *D. elegans* and *D. rhopaloa*. (Figure S2 and Table 2). These euchromatic copies are difficult to detect cytologically on mitotic chromosomes due to their lower copy number and lack of compaction as compared to copies within heterochromatin. Therefore, to confirm the presence of *1.688* satDNA arrays in euchromatic regions, FISH experiments were performed on polytene chromosomes from the salivary glands of nine species (Figure 6). Polytene chromosomes undergo several rounds of endoreduplication in their euchromatic regions, but the centromeric heterochromatin does not endoreduplicate and instead forms a dense central mass known as the chromocenter (Riddle *et al.* 2011). Therefore, FISH on polytene chromosomes enables both the detection of low-copy number targets due to their amplification as well as the confirmation that they are located within the euchromatic regions of the chromosomes. Our FISH analysis showed several hybridization signals throughout the euchromatic regions for all nine species (Figure 6). We detected the presence of *1.688 satDNA* repeats within euchromatic regions of the X, 2^nd^ and 3^rd^ chromosomes in different species, but no signals were detected on the 4^th^ and Y chromosomes. Notably, we detected strong signals on the telomeric portions of *D. eugracilis* and *D. biarmipes* chromosomes (Figure 6 J-K, respectively), confirming and expanding previous findings by Saint-Leandre *et al.* (2019).

To identify possible preferential euchromatic locations, we ran BLAST searches using a species-specific euchromatic consensus sequence as a query in the available genome assemblies of *D. melanogaster*, *D. simulans* and *D. yakuba*. The locations of the euchromatic *1.688* satDNA varied significantly throughout the chromosomes of the three species and, consistent with our FISH analyses, we did not detect copies on the fourth and Y chromosomes. *D. yakuba* showed the highest presence of euchromatic arrays, followed by *D. melanogaster* and *D. simulans*. (Table S3). Furthermore, euchromatic repeats are enriched on the X chromosome in all three species (Table S3). In *D. melanogaster* and *D. simulans* the majority of euchromatic arrays are on the X chromosome (83% and 80%, respectively). In contrast, *D. yakuba* has only 61% of *1.688* satDNA arrays localized on the X chromosome (Table S3). To further understand the genomic distribution of the euchromatic repeats in these three species, we analyzed their density throughout each chromosome and found that each species showed a unique pattern of localization (see Methods; Figures S5-9). Altogether, *1.688*-like satDNA sequences in euchromatic regions is a common feature of *Drosophila* genomes within the *D. melanogaster-D. rhopaloa* clade. We suspect the species-specific distribution pattern occurred during the initial phases of the process of speciation, with differential expansions through recurrent events of mobilization (Sproul and Khost *et al.* 2020).

### Euchromatic repeats display high nucleotide divergence in intra- or interspecific comparisons

**Figure 7:**
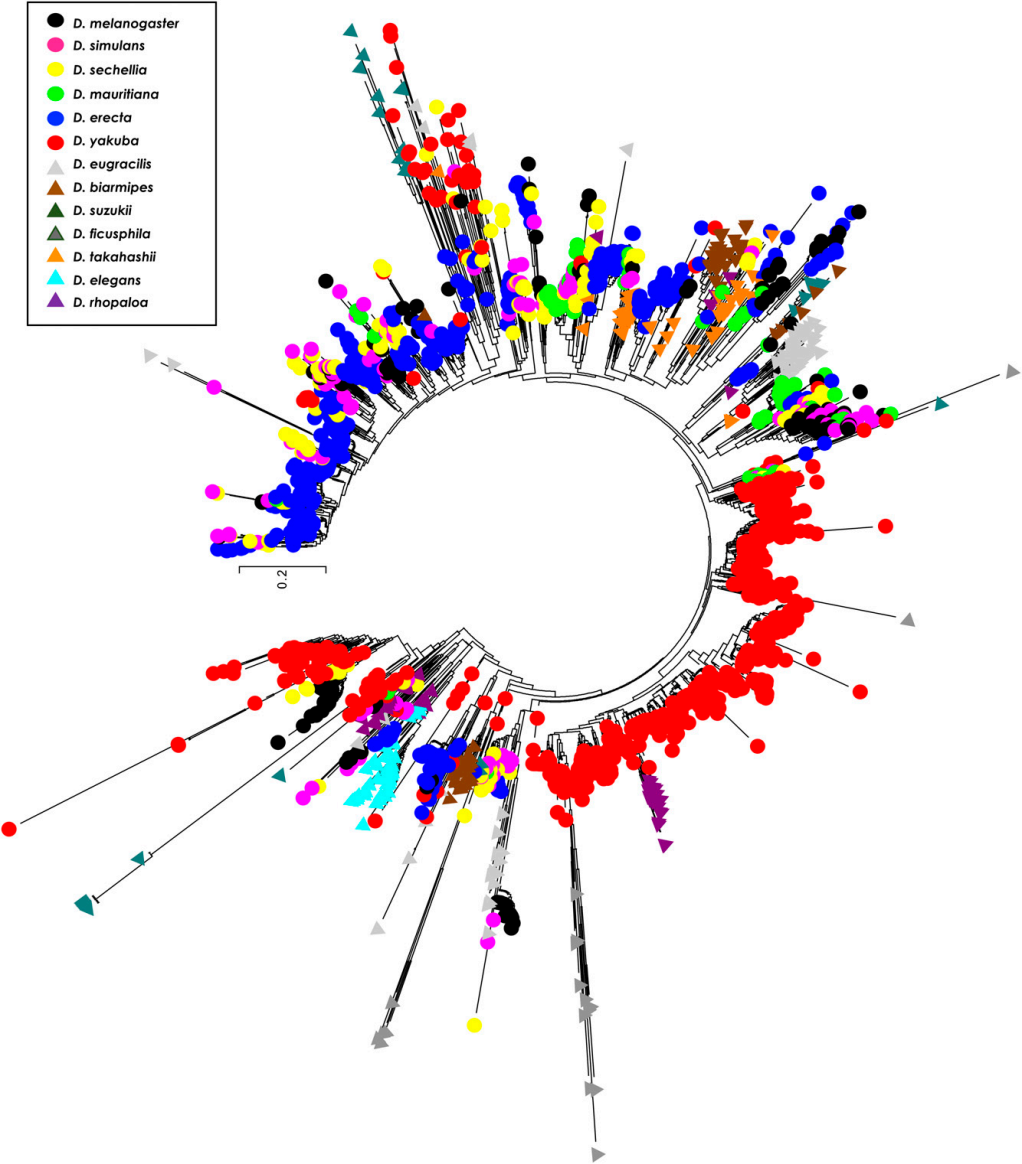
Lack of species-specific clusterization (except in *D. yakuba*) of euchromatic copies of *1.688* satDNA indicates broad interspecies divergence. An unrooted phylogenetic tree using 2393 full-length *1.688-like* monomers derived from euchromatin from 13 species of *Drosophila* shows intermingling throughout *melanogaster group* phylogeny. *D. yakuba* is a notable exception in which the species-specific branch indicates a recent expansion of 190 bp repeats in this species. The evolutionary history was inferred using methods and parameters described in Figure 3.

Similar to our analysis of heterochromatic repeats, we ran an NJ phylogenetic analysis with all 2,393 full-length repeats retrieved from 13 species. We excluded *D. orena* from this analysis because the euchromatic copies we identified were mostly truncated or partial when compared to heterochromatic copies. The tree topology showed large branch lengths with three major groups (Figure 7). One branch is composed almost exclusively of monomers from the 190 bp satDNA subfamily in *D. yakuba*, indicating a high degree of sequence divergence of this variant within this species. The two other branches do not show any consistent phylogenetic grouping, and sequences derived from the same arrays do not cluster predominantly together. Further, a species-specific analysis of the euchromatic arrays of *D. melanogaster*, *D. simulans*, *D. sechellia*, *D. erecta* and *D. yakuba* failed to show a higher frequency of clusterization of *1.688* satDNA sequences positioned on the same chromosome when compared to arrays located on non-homologous chromosomes, although some branches appear to show array-specific or species-specific patterns. Our analysis of the intra- and interspecific nucleotide divergence of the euchromatic *1.688* satDNA copies showed a broad range of variation (Table 4). Intraspecific nucleotide divergence ranged from 14.2% in *D. elegans* to 43.3% in *D. ficusphila*, and overall, we observed a higher degree of divergence for *1.688* satDNA euchromatic copies (23.7% on average) than heterochromatic copies (13.2% on average), indicating euchromatic copies tend to be highly divergent within a species. Together, the patterns of clusterization and nucleotide divergence indicate that *1.688* satDNA euchromatic and heterochromatic sequences experience different evolutionary pressures.

**Table 4:** Intra- and interspecific nucleotide divergence (*p*-distance) of 2,393 *1.688* satDNA euchromatic copies retrieved from 13 species of *Drosophila*

*D. mel*	*D. sim*	*D. sec*	*D. mau*	*D.ere*	*D.yak*	*D.eug*	*D.bia*	*D.suz*	*D.tak*	*D.fic*	*D.ele*	*D.rho*
0.274												
0.273	0.233											
0.276	0.24	0.239										
0.261	0.236	0.247	0.185									
0.269	0.229	0.229	0.242	0.198								
0.284	0.252	0.253	0.231	0.239	0.176							
0.325	0.303	0.309	0.297	0.29	0.297	0.282						
0.303	0.279	0.284	0.265	0.27	0.274	0.319	0.18					
0.397	0.377	0.378	0.377	0.361	0.361	0.408	0.375	0.369				
0.261	0.239	0.245	0.217	0.227	0.233	0.285	0.233	0.369	0.18			
0.409	0.387	0.389	0.388	0.379	0.373	0.443	0.423	0.46	0.378	0.433		
0.335	0.314	0.318	0.3	0.312	0.3	0.358	0.297	0.398	0.286	0.411	0.142	
0.285	0.267	0.268	0.25	0.249	0.235	0.316	0.269	0.364	0.238	0.381	0.286	0.196

### 1.688-Like elements are present in the vicinity of genes of melanogaster subgroup species

Due to the pervasive localization of *1.688* satDNA arrays in euchromatin, we analyzed whether *1.688* satDNA insertion sites are present near gene regions. We ran species-specific BLASTN searches in the promoter/upstream, coding, intronic and downstream regions of all genes annotated in the UCSC *GenomeBrowser* (www.genome.ucsc.edu) for the five species of the *melanogaster* subgroup that have annotated genomes (*D. melanogaster*, *D. simulans*, *D. sechellia*, *D. erecta* and *D. yakuba*). Using stringent query parameters to prevent non-specific hits, we identified both full and partial *1.688* satDNA arrays in proximity to genic regions (maximum of 5 kb) or within introns (See Methods). From all five species, we found 921 insertions in the promoter/upstream regions, 825 insertions in downstream regions, and 572 insertions in intronic regions (Supp. Mat. 3).

**Figure 8:**
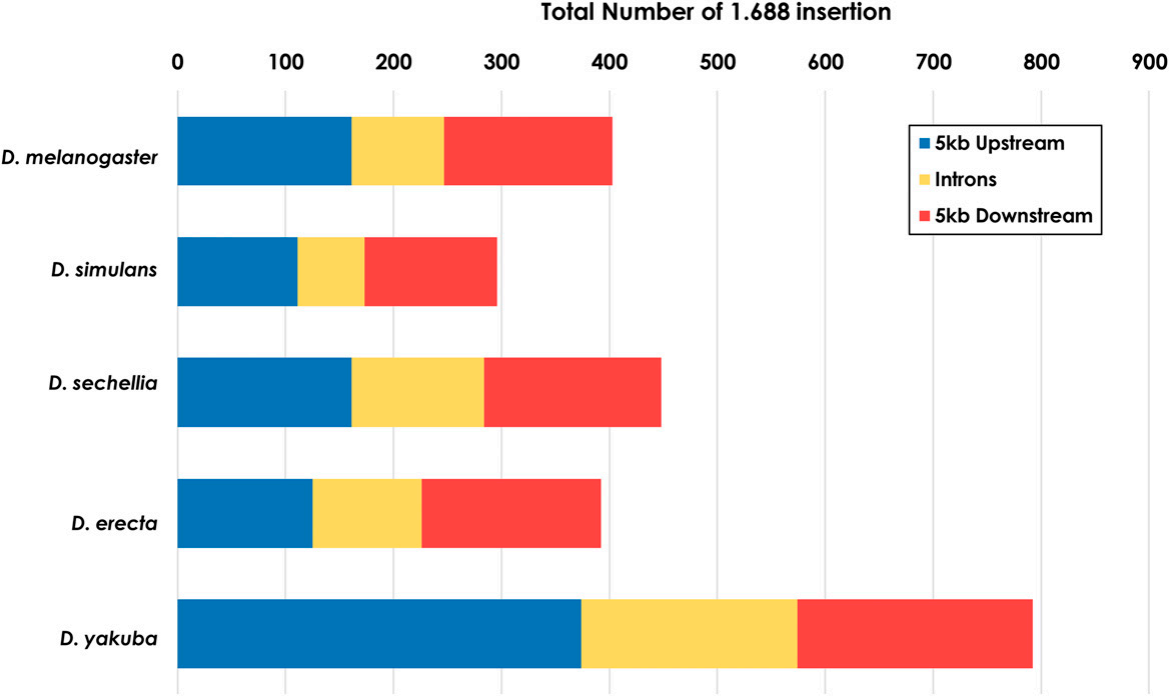
Insertions of *1.688* satDNA sequences in the vicinity of genes. Species-specific BLASTN searches in the upstream, intronic and downstream regions of all annotated genes from the UCSC *GenomeBrowser* (www.genome.ucsc.edu) was performed for the five species of the *melanogaster* subgroup with annotated genomes.

The number of insertions varied in the different types of regions and are not well conserved among the species (Figure 8). *D. yakuba* has the largest number of insertions with 374, 218 and 200 instances in upstream, downstream and intronic regions, respectively. In *D. simulans*, we identified 111 *1.688* satDNA sequences present in upstream regions, 123 in downstream regions and 62 in intronic regions. *D. sechellia* and *D. erecta* showed similar distribution patterns in the three regions (Figure 8). *D. melanogaster* has the second lowest number of gene proximal insertions in all five species with 149, 156 and 86 insertions in upstream, downstream and intronic regions, respectively. Altogether, we find that *1.688* satDNA sequences are not only present in euchromatic regions but can be in close proximity to genes, with each species displaying a unique and dynamic pattern throughout euchromatin.

### The intronic insertion of 1.688 satDNA within Netrin paralogs is evolutionarily conserved

**Figure 9:**
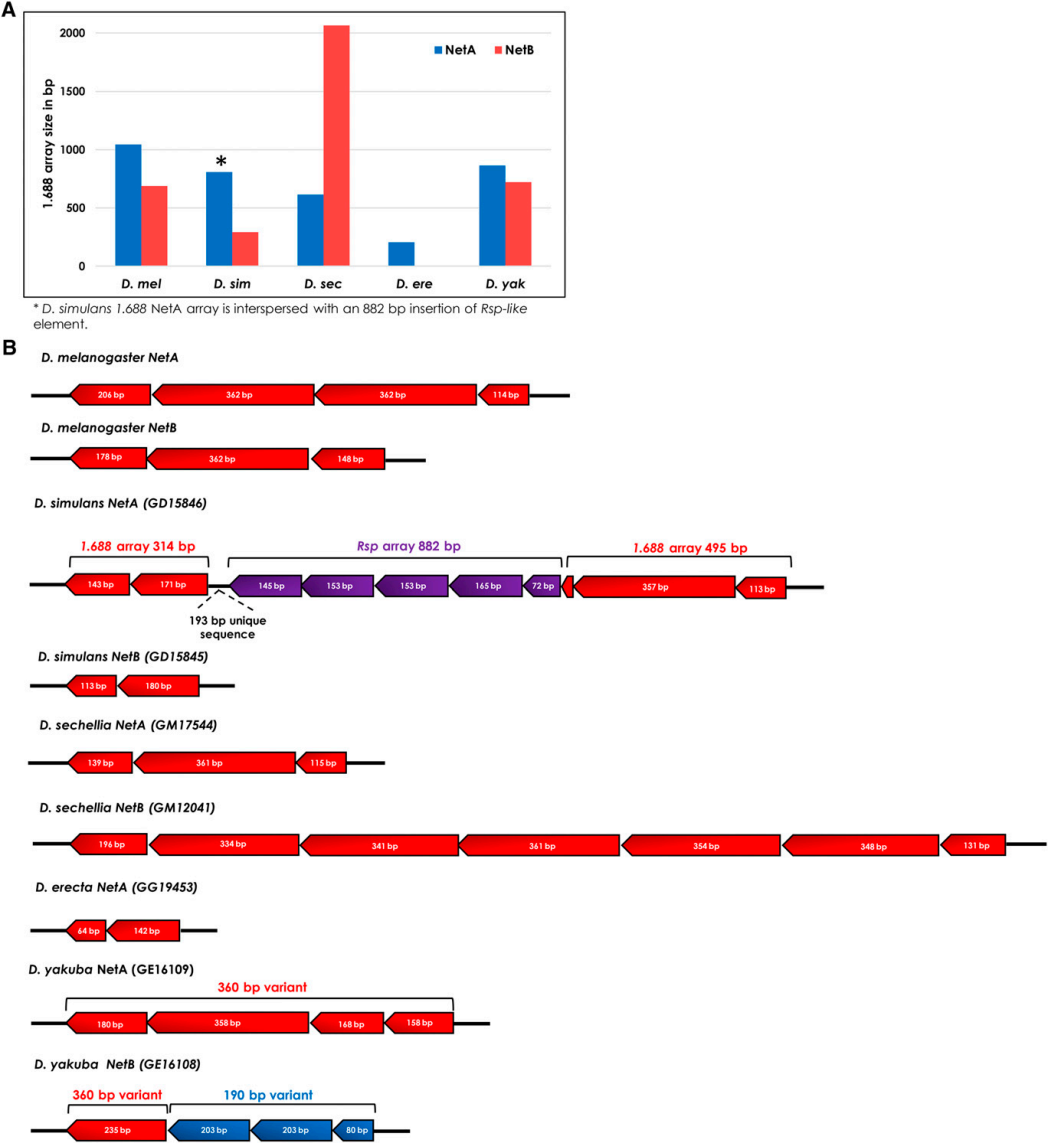
Differential pattern of *1.688*satDNA insertions in the intronic regions of duplicated genes *NetA* and *NetB* in five species of the *melanogaster* subgroup. A. Histogram representation of *1.688* array size insertion in the intronic regions of *NetA* and *NetB* genes, respectively, for five species of *melanogaster* subgroup. B. Schematic representation of *1.688* insertion in the intronic regions of *NetA* and *NetB* genes in five species of *melanogaster* subgroup evidencing a species-specific pattern of *1.688* array size and organization. The 360 bp subfamily is represented in red, whereas the 190 bp subfamily is in blue. *Responder-like* arrays are represented in purple. Note that repeat units sizes identified here are based on the monomeric sequence designed for each species.

A striking example of an evolutionarily conserved insertion site is the intronic regions of the duplicated gene *Netrin*. The products of *NetA* (Dmel: FBgn0015773) and *NetB* (Dmel: FBgn0015774) are crucial instructive cues for target recognition at the fly neuromuscular junction (Orr *et al.* 2014). The genes are in tandem on the X chromosome and contain a *1.688* satDNA array in the intronic region in all five species with annotated genomes except *D. erecta*, which has the shortest *1.688* satDNA array in *NetA* and no array in *NetB* (Figure 9A). Surprisingly, in *D. yakuba* the *NetA* array carries the 360 bp variant whereas *NetB* array carries the 190 bp variant as identified by its deletion signature (Figure S9B). In other species, only the 360 bp variant is present in the *NetA* and *NetB* introns. This pattern of evolutionary conservation suggests that the *1.688* satDNA insertion likely occurred prior to the gene duplication event, which was before the speciation of the *melanogaster* subgroup. Interestingly, the *1.688* satDNA monomeric sequences present in the *NetA* and *NetB* introns do not evolve neutrally. Although the *NetA* coding sequence appear to have evolved under neutral selection (*D*=-0.09, Tajima’s test), the *1.688* satDNA sequences inserted in *NetA* and *B* introns, and the *NetB* coding sequence, all show signs of positive selection (*D*=-0.53, -0.43, -0.74, respectively). This finding suggests that *1.688* satDNA euchromatic insertions in the vicinity of genes could play a role as a *cis*-acting gene regulation element.

Additionally, we found an insertion of 882 bp of *Responder*-like satDNA exclusively inside the *NetA 1.688* satDNA array in *D. simulans* (Figures 9B and S10A). This insertion also carries a 193 bp sequence highly similar to the intronic region of the *Flotillin 2* gene (Dsim: *FBgn0068599*) and an adjacent *1.688* satDNA array(Figure S10). The same 193 bp sequence is observed in the orthologous sites of closely related species from the *melanogaster* subgroup (Figure S10A). In *D. simulans*, the high sequence identity between the 193 bp sequence present at *Flotillin 2* and *NetA* (98.4% of identity), together with the nearly identical 233 bp flanking fragment of *1.688* on both gene introns (96.2% of identity) (Figure S10B) and the absence of this insertion in the close related species *D. sechellia* suggests a recent transposition event of a *Responder-like* element from *Flotillin 2* to *NetA* in which a partial sequence of the *1.688* satDNA array was carried during this element mobilization. Finally, the *Responder*-like element transposition from one *1.688* satDNA array to another reinforces the hypothesis that the evolution and proliferation of both satDNA families in euchromatin are co-dependent (Sproul and Khost *et al.* 2020).

## Discussion

In the present study we performed a large-scale analysis of the *1.688* satDNA family in genomes from 16 species in the *melanogaster* group utilizing a combination of assembled genomes, *de novo* repeat identification, similarity estimation methods and molecular cytogenetic analyses. Our report greatly expands the knowledge regarding the prevalence and evolution of *1.688* satDNA sequences in the *melanogaster* group of species (Figure 1; Figure 4). Additionally, our data are consistent with previous observations that *1.688* satDNA sequences are not confined to heterochromatic regions but are also present in euchromatin, which we demonstrate for the first time in *D. erecta*, *D. yakuba*, *D. eugracilis*, *D. biarmipes*, *D. suzukii and D. takahashii* (Figure 6) and predict in *D. ficusphila*, *D. elegans* and *D. rhopaloa* (Figure S2). Our data suggest that *1.688* satDNA amplification probably occurred in the ancestral lineage of the *D. melanogaster-D. rhopaloa* clade after the divergence from the common ancestor shared with the *montium* subgroup of species ∼27 Mya. Alternatively, the absence of *1.688* satDNA copies in *D. kikkawai* and *D. leontia* may derive from stochastic processes or concerted evolution that have led to significant nucleotide divergence, which may hinder identification in these species. The maintenance of conserved satDNA sequences can indicate functional constraints, such as the centromeric satDNA in primates (Alkan *et al.* 2007). However, in *Drosophila* the long-term evolutionary maintenance of satDNA is rare. Previous analyses have described the maintenance of the satDNA family *pvB370* for a period of about 20 My in the *D. virilis* group species, with varying abundance in each species (Heikkinen *et al.* 1995; Biessmann *et al.* 2000). To our knowledge, this was the most broadly maintained satDNA family until our description of *1.688* satDNA as a genomic feature of 16 species in *melanogaster* group. Although *1.688* satDNA has been maintained for an exceptionally long period of time, it also exhibits great variability in genomic proportions, varying from 0.091% in *D. biarmipes* to 4.345% in *D. rhopaloa* (Figure 1). Recurrent turnover events of different satDNA families, such as *Rsp*, *dodeca*, and simple satellite DNAs, along with heterogeneity in chromosomal localization of satDNA families has been described in *Drosophila* species (Larracuente 2014; Carmena *et al.* 1993; Jagannathan *et al.* 2017; Wei *et al.* 2018), reinforcing that satDNA evolution is dynamic and can impact the genomic landscape of a species It is important to highlight that the two satDNA families with broader coexistence patterns throughout *Drosophila* phylogeny, *pvB370* and *1.688*, were previously described on both domains of chromatin (Biessmann *et al.* 2000 and herein). satDNA in heterochromatin turns over faster than in euchromatin, so its presence in euchromatin may aid its evolutionary maintenance.

Computational and experimental evidence both support differences in the abundance and location of the *1.688* satDNA family throughout the *melanogaster gro**up* phylogeny (Figures 4 and 5). However, the presence of *1.688* satDNA arrays on the telomere proximal region of chromosome X in six out of nine species analyzed (Figure 4 and 5) suggests that this *1.688* satDNA block may be evolutionarily conserved and leads us to speculate that this particular location may be the ancestral position. We demonstrate that *1.688* heterochromatic arrays are present at different locations and on different chromosomes, even in closely related species (Figure 5), indicating the spread of heterochromatic *1.688* satDNA may have followed unique trajectories in each lineage. A striking example is shown by the dramatic differences in chromosome localization of the *1.688* satDNA heterochromatic arrays in *D. simulans* and *D. sechellia* (Figure 5), which is in spite of the monomers lacking a clear species-specific cluster or significant nucleotide divergence (Figure 3; Table 3). These findings suggest that even though satDNAs have a high rate of evolution in eukaryotic genomes (Charlesworth 1994), *1.688* satDNA sequences do not necessarily evolve as rapidly as their location changes, at least in the heterochromatin of these species. Overall, our findings highlight a paradoxical evolutionary characteristic of the *1.688* satDNA family: persistence over a long evolutionary period with species-specific patterns of location and sequence variation.

In addition to heterochromatic arrays, *1.688* satDNA euchromatic copies can contribute significantly to the genome content of the *melanogaster* group, especially in *D. yakuba* (Figure S2). Notably, *1.688* satDNA euchromatic arrays are enriched on, but not restricted to, the X chromosome (Figure 6, Table S3). The enrichment of *1.688* satDNA in the euchromatic portions of the X chromosome may be related to specific sex-chromosome characteristics (Gallach 2014) or to functional constraints, as X-linked transcripts derived from *1.688* satDNA are reported to act as siRNAs that enhance the dosage compensation by MSL complex proteins (Menon *et al.* 2014). The exact mechanisms of colonization of different heterochromatic/euchromatic regions is still unknown. However, a recent study suggests that *Rsp*-dependent transposition by extrachromosomal DNA may mobilize and spread *1.688* satDNA sequences (Sproul and Khost *et al.* 2020). Therefore, we speculate that *1.688* satDNA repeats were established in euchromatin early in the evolution of the *melanogaster* group and subsequent unique mobilization and diversification events contributed to the species-specific patterns observed.

The species-specific patterns of *1.688* satDNA repeats in heterochromatin (Figure 3) contrasts with the lack of array-specific or chromosome clusterization for most repeats in euchromatin (Figure 7). Taken together, these observations suggest the operation of different molecular forces in the two types of chromatin. The concerted evolution of repeats in heterochromatin indicates molecular drive mechanisms (Dover 1986). Conversely, recombination in repeated regions may be repressed in euchromatin since unequal exchanges may have negative consequences such as gene deletions (Feliciello *et al.* 2006). Our evidence that *1.688* satDNA sequences display different evolutionary signatures depending on the chromatin context suggests that they may be subject to different molecular processes, leading us to speculate that the differential location of *1.688* satDNA sequences in heterochromatin and euchromatin may shape the sequence landscape of the *1.688* satDNA family in each species.

Although the most prevalent *1.688* satDNA monomer size is ∼360 bp (Carlson and Brutlag 1977), the 190 bp variant is present throughout *D. melanogaster* group phylogeny (Table 2). First described in the *D. teissieri* genome (Strachan *et al.* 1985), our data indicate that this variant is pervasive and can comprise the majority of copies in some species, such as in *D. ficusphila* and *D. suzukii*. The prevalence of the 360 bp and 190 bp variants throughout the *D. melanogaster-D.rhopaloa* clade as described here leads us to conclude that both were likely present in the last common ancestor ∼27 Mya, and while it is impossible to know which variant is the most ancestral, we favor a model where the 360 bp variant gave rise to the 190 bp variant through some recombination event during the evolutionary history of this clade. Notably, the 190 bp variant in *D. suzukii* does not share the same structure observed in the rest of the species (Figure 2). This result suggests that 190 bp subfamilies either passed through a species-specific homogenization event, or that an independent event generated the 190 bp variant, as we hypothesize for the *D. suzukii* 190 bp variant. Interestingly, the length of both *1.688* satDNA subfamilies (190 and 360 bp) corresponds to the length of DNA wrapped around 1 or 2 nucleosomes, respectively (Henikoff *et al.* 2001), suggesting that the maintenance of both monomer sizes could be linked to this genomic architecture feature.

*D. yakuba* has the largest number of euchromatic *1.688* satDNA copies out of all 14 species we analyzed (Figure S2, Table 2). Due to the species-specific pattern of their clustering (Figure 7), we speculate there was a relatively recent expansion by lineage-specific events such as rolling-circle amplification and insertion (Sproul and Khost *et al.* 2020). It is noteworthy that different mechanisms, such as gene conversion, may also perform important roles in the expansion of the 190 bp subfamily, as observed in *NetB* repeats (Figure S10). Intriguingly, *D. yakuba* also has the largest number (28) of fixed chromosomal inversions identified in *Drosophila* (Ranz *et al.* 2007), the breakpoint of which are described as hotspots for repeat insertions (Casals *et al.* 2006). Therefore, we suggest that the high number of *1.688* satDNA euchromatic insertions in *D. yakuba* may reflect genomic instability events that occurred during the evolution of this species.

*1.688* satDNA arrays are present at telomeric regions in *D. biarmipes* and *D. eugracilis* genomes, suggesting that this satDNA family may have been co-opted to perform the telomeric function in these species. *Drosophila* telomeric regions are composed of transposable elements (Het-A/TART) responsible for maintaining telomeric sequences and a protein complex (HOAP) required for telomere stability (Silva-Sousa and Casacuberta 2012). A recent study by Saint-Leandre *et al.* (2019) failed to identify the expected telomere-specialized elements in the *D. biarmipes* genome and suggested that telomeric function relies on euchromatic *D. melanogaster 1.688* satDNA repeats (also known as SAR2 sequences) and *Helitron-like* transposable elements. *Previous studies have shown that D. melanogaster satDNA sequences interact with recombinant HOAP complex proteins (**Shareef et al. 2001**)*. Therefore, the presence of *1.688* satDNA in the telomeric regions of *D. biarmipes* and *D. eugracilis* combined with the apparent absence of Het-A and TART sequences (in *D. biarmipes*) suggests that *1.688* satDNA may serve a telomeric function in these two species.

Finally, we found evidence for insertions of *1.688*-like satDNA elements in gene proximal regions in *melanogaster* subgroup species (Figure 8). This distribution indicates that at least at some positions, gene-proximal insertions are tolerated (Feliciello *et al.* 2015). Furthermore, the insertions in the intronic regions of *NetA* and *NetB* genes show evidence of positive selection. Previous studies proposed that *1.688* satDNA sequences may act as *cis*-regulatory elements, such as promoters, insulators or transcription factor binding sites (Menon and Meller 2015). Thus, the presence of 921 *1.688* satDNA hits located on the 5 kb upstream regions of genes for the five species analyzed suggests that *1.688* satDNA repeats might act as local modulators of transcription, as observed for transposable elements inserted immediately upstream of protein coding genes (Faulkner *et al.* 2009). The transcriptional potential of *1.688* satDNA (Menon *et al.* 2014), as well its distribution near protein-coding genes, supports the hypothesis that *1.688* satDNA may represent a rich source for the assembly of gene regulatory systems (Menon and Meller 2015). Moreover, Ekhteraei-Tousi *et al.* (2020) have recently shown that the depletion of the *1.688* satDNA block within chromosome X pericentromeric regions may influence the differential expression of genes involved in eggshell formation, and that the transcripts from these regions may act in *trans*. Much remains to be done to understand the influence of *1.688* satDNA sequences on the transcriptional landscape. However, it is tempting to speculate that the disparate pattern of *1.688* satDNA insertions close to protein-coding genes, along with its unique diversification in *Drosophila* species, could contribute to the establishment of lineage-specific or species-specific patterns of gene expression.

In summary, our work represents the most comprehensive analysis of *1.688* satDNA evolution to date, demonstrating that this satDNA family likely originated after the divergence of *D. melanogaster-D. rhopaloa* and *montium gro**up* species (27 Mya). The locations of *1.688* satDNA in genomes throughout *Drosophila* phylogeny, and the presence of conserved sequence motifs, suggests *1.688* satDNA may provide a “multi-use tool” function for chromosome biology, consistent with roles at centromeres, telomeres, and in regulating gene expression. Our findings lay a strong foundation for future studies aimed to better understand the biological roles of *1.688* satDNA sequences, and satDNAs in general, during the evolutionary history of *Drosophila* species.
